# Socio-ecological influences on adolescent (aged 10–17) alcohol use and linked unhealthy eating behaviours: protocol for a systematic review and synthesis of qualitative studies

**DOI:** 10.1186/s13643-017-0574-8

**Published:** 2017-09-02

**Authors:** Stephanie Scott, Jessica Reilly, Emma L. Giles, Frances Hillier-Brown, Louisa Ells, Eileen Kaner, Ashley Adamson

**Affiliations:** 1 Institute of Health & Society, Baddiley-Clark Building, Newcastle University, Richardson Road, Newcastle upon Tyne, NE2 4AX UK; 2 Health and Social Care Institute, 1.18 Constantine Building, Teesside University, Borough Road, Middlesbrough, TS1 3BX UK

**Keywords:** Adolescent, Eating, Alcohol use, Qualitative research, Systematic review

## Abstract

**Background:**

Excess body weight and risky alcohol consumption are two of the greatest contributors to global disease. Health behaviours cluster in adolescence and track to adulthood. Very little is known about similar and contrasting influences on young people’s eating behaviours and alcohol use. Whilst there are bodies of literature which explore the influences on young people’s eating behaviour and alcohol consumption respectively, no qualitative studies have been identified with an explicit and concurrent focus on adolescent eating behaviours and alcohol consumption. This review will identify and synthesise qualitative research evidence to provide insight into common underlying factors which influence alcohol use and unhealthy eating behaviours amongst young people aged 10–17. This will involve bringing together two separate bodies of literature to enable analysis and comparison across two associated fields of study.

**Methods:**

We will conduct searches in MEDLINE, Scopus, PsycINFO, Sociological Abstracts (via ProQuest social science premium collection), CINAHL, ERIC, IBSS (via ProQuest social science premium collection), ASSIA (via ProQuest social science premium collection), and Web of Science Core Collection. Studies reporting primary data of any qualitative design, for example, ethnographic studies, studies that used a phenomenological or grounded theory approach, or participatory action research will be included in the review. Database searches will be supplemented with searches of Google Scholar, hand searches of key journals, and backward and forward citation searches of reference lists of identified papers. Search records will be independently screened by two researchers, with full text copies of potentially relevant papers retrieved for in-depth review against the inclusion criteria. Reporting of identified studies will be assessed using the Critical Appraisal Skills Programme (CASP) Qualitative Research Checklist. GRADE-CERQual will also be used to assess confidence in the findings arising from our review. Qualitative synthesis will involve three core phases: line-by-line coding of findings; development of descriptive themes; and development of analytical themes. Findings from papers will be examined for overlaps, similarities and differences.

**Discussion:**

This synthesis will interpret individual studies by identification of second-order constructs (interpretations offered by the original researchers) and third-order constructs (development of new interpretations beyond those offered in individual studies) by way of the development of a ‘model structure’ of shared influences upon both unhealthy eating behaviours and alcohol use. It is anticipated that this ‘model structure’ will aid subsequent co-design and piloting of a future intervention to help reduce health risk and social inequalities due to excess weight gain and alcohol consumption.

**Systematic review registration:**

CRD42017060624.

**Electronic supplementary material:**

The online version of this article (10.1186/s13643-017-0574-8) contains supplementary material, which is available to authorized users.

## Background

Excess body weight and heavy alcohol consumption are two of the greatest contributors to global disease burden in high-income countries [1, 2] and are amongst the most well-established preventable causes of cancers including breast, liver, kidney, bowel, mouth, throat, and prostate [3, 4]. Being overweight and/or obese accounts for 5% of deaths worldwide [2] and is responsible for raising the risk of chronic diseases such as diabetes and cardiovascular diseases [5]. Heavy alcohol consumption contributes to over 200 disease and injury conditions and is responsible for almost 6% of world deaths [1]. Risky or heavy alcohol use is the leading cause of death and disability adjusted life years in both 15–19-year-olds and 20–24-year-olds globally [6]. Rates of liver disease are linked to both alcohol use and obesity and they are rising rapidly in the UK [7, 8], particularly in those aged below 44 years [7]. Over the past 30 years, the UK has seen a fourfold increase in liver disease mortality, and it is now the third most common cause of premature death, with 62,000 years of working life lost each year [9]. Most of these deaths are alcohol-related [10]. However, non-alcohol fatty liver disease (NAFLD) is becoming increasingly common, and it is now the most prevalent liver disorder in children and young adults with overall prevalence of between 2.6 and 9.8% in over-weight individuals which rises to between 42 and 77% in those who are obese [9]. In addition, it has been shown that the combination of a raised body mass index (BMI) and heavy alcohol consumption can result in an intensified interaction creating a steeply elevated risk of liver disease in men and women [11] and that heavy drinking is associated with greater waist-hip ratio in mid-life even when taking other lifetime influences into account [12].

Research on reducing excess body weight or heavy alcohol consumption typically occurs in isolation, or as part of non-specific multiple behaviour change interventions. However, strategies to jointly reduce alcohol consumption and address levels of overweight or obesity may produce greater health gains, and be a more efficient use of resources, than initiatives directed towards each pattern alone [11]. As such, understanding the relationship between these behaviours in order to develop intervention pathways is a global public health priority [11], particularly amongst younger people [13]. Adverse health behaviours begin to cluster during adolescence [14], and excess body weight and risky drinking have both been demonstrated to track into and throughout adulthood [15–17]. Consequently, there is a strong rationale to intervene with young people early, before these behaviour patterns become fully entrenched habits which lead to poorer health and social outcomes later in life.

Many eating rituals have become strongly linked to the use of alcohol and vice versa, for instance salty snacks are often sold in public drinking venues and there is a popular concept of drinking alcohol with dinner, eating and drinking at parties or social gatherings and visiting fast food outlets after an evening out at drinking establishments [18]. Indeed, it has been suggested that unhealthy food choices are more likely to be made during and directly after a period of prolonged alcohol consumption [18] which could be due, at least in part, to the disinhibiting effect of alcohol which is a psychoactive substance that can alter usual behaviour. However, there are key differences when thinking about eating and unhealthy drinking behaviour. All individuals need to eat to survive, whilst many individuals choose to drink alcohol because it is perceived to be a pleasurable component of social life. Further, whilst alcohol contains energy, it is a nutritionally poor food source and does not stimulate satiety [19]. This may make it more likely for alcohol calories to be consumed in addition to energy intake from food.

Epidemiological data suggest that energy intake from alcohol, type of beverage and drinking pattern (i.e. high volume, high frequency) are associated with excess body weight and weight gain amongst adults [19, 20]. Few studies have explored this relationship amongst young people. Those that do are predominantly quantitative and conducted with young adults (18+) in US college settings. These studies have shown a positive association between being overweight and/or obese and alcohol consumption, particularly amongst females [21, 22], and highlighted a conflict for some individuals between a wish to stay slim and also to drink alcohol as part of developing a social identity [23–25]. Furthermore, there have been some reports of individuals choosing not to eat prior to socialising, so that they can drink alcohol and avoid weight gain; a phenomenon that has been termed ‘drunkorexia’ [23]. This increases the likelihood of intoxication, where blood alcohol levels rise sharply and affect the brain and subsequent behaviour, which steeply increases the risk of acute harm from drinking.

Little more is known about young people’s (under the age of 18) perspectives on the relationship between alcohol consumption and unhealthy eating behaviour, i.e. patterns of food choice or behaviours that lead to adverse health outcomes, such as snacking, eating energy-rich or high-sugar foods or avoiding eating so that alcohol calories do not lead to weight gain. It is increasingly recognised that health-promoting interventions must acknowledge social and emotional needs [26, 27] as well as focus on reducing health risks. Recent work has begun to investigate wider socio-cultural drivers of drinking and eating behaviour in early adolescence [28, 29] but this needs to extend to an understanding of co-occurring health behaviours in adolescence and early adulthood. Whilst there are bodies of literature which explore the influences on young people’s eating behaviour and alcohol consumption respectively, scoping searches of ProQuest, Web of Science, the Joanna Briggs Institute (JBI) Library, Cochrane Library and PROSPERO identified no qualitative studies with an explicit and concurrent focus on adolescent eating behaviours and linked alcohol consumption and no existing systematic reviews on this topic. There are emotional, social and symbolic benefits of health risk behaviours for young people, which appear to cut across both food and alcohol consumption practices including perceptions of pleasure, distinction and identity or social status [26, 27, 30–32]. Further, food and alcohol consumption takes place within a wider social context which can adversely shape behaviour. These are sometimes described as the ‘foodscape’ or ‘obesogenic’ and ‘intoxigenic’ environments, where physical, urban spaces come together with social, cultural and commercial influences to shape behaviour but are not always consciously recognised as doing so [33–39]. Interpersonal ties, such as peer and family networks, have also been shown in qualitative studies to influence both alcohol use and eating behaviour [40–43], likewise socio-economic status and austerity [44–46].

Most existing reviews answer a very specific question, such as alcohol industry efforts to influence alcohol marketing policy [47], or synthesise quantitative rather than qualitative research, or a combination of the two [48, 49]. Therefore, it was not deemed appropriate to synthesise from these reviews as a ‘review of reviews’. Thus, this synthesis will involve bringing together two separate bodies of literature in order to make inferences about factors that might influence people amongst whom these behaviours co-occur. In doing so, it is our intention to capitalise on what is known from independent streams of research, enabling analysis and comparison across two associated fields of study. Guided by the approach suggested by the EPPI-Centre (IoE, London), the systematic review proposed here will address this evidence gap by reviewing and synthesising qualitative research evidence to provide insight into common underlying factors which influence alcohol use behaviour and eating behaviour amongst young people aged 10–17. Relevant qualitative synthesis techniques, an approach increasingly adopted in reviews of qualitative health research, will be used to develop a model structure, or typology, of overlapping and contrasting influences across both consumption behaviours, which can be used to inform subsequent exploratory work and co-design of tailored, dual-focused interventions with young people.

### Research question

Our primary objective is to examine young people’s (aged 10–17) perspectives on socio-cultural, interpersonal and structural influences upon unhealthy eating behaviours or alcohol use by synthesising data from qualitative research evidence, in order to make inferences about factors that might influence people amongst whom these behaviours co-occur and to develop a model structure of common underlying influences which cut across unhealthy eating behaviours and alcohol use amongst young people.

## Methods

### Study registration

The review will be carried out following established criteria for the good conduct and reporting of systematic reviews [50] and reporting will adhere to Preferred Reporting Items for Systematic Reviews and Meta-Analyses (PRISMA) statement guidelines [51]. The review will be structured according to the reporting guidelines for synthesis of qualitative studies (the ENTREQ statement) [52]. This protocol was structured according to the Preferred Reporting Items of Systematic Reviews and Meta-Analyses Protocol (PRISMA-P) guidelines [53] (see Additional file 1) and is registered with the PROSPERO International Prospective Register of Systematic Reviews (Ref: CRD42017060624).

### Search strategy

The search strategy has been split into five core concepts in accordance with the SPIDER tool [54] which is deemed an appropriate approach for qualitative evidence reviews (see Table 1). The concepts of ‘design’ and ‘research’ type will be combined to maximise yield of qualitative literature. A three-step search strategy will be undertaken. A scoping search of MEDLINE and CINAHL will be used to identify keywords and phrases in the paper title and abstract and MeSH/thesaurus terms used to index relevant articles. A second search using identified keywords and thesaurus terms will subsequently be undertaken across all included databases. Thesaurus terms will be translated and truncated as appropriate across databases. The following databases will be searched: MEDLINE, Scopus, PsycINFO, Sociological Abstracts (via ProQuest social science premium collection), CINAHL, ERIC, IBSS (via ProQuest social science premium collection), ASSIA (via ProQuest social science premium collection), and Web of Science Core Collection.

**Table 1:** Review concepts and associated search terms

SPIDER concept	Search terms
S—Sample: young people	Child OR children OR adolescent OR youth OR young people OR young person OR young adult OR kid OR teenager OR under-age OR student
PI—Phenomenon of interest: alcohol consumption OR unhealthy eating behaviours	Alcohol drinking OR alcoholic beverages OR alcoholic intoxication OR alcohol consumption OR alcohol misuse OR alcohol abuse OR alcohol use OR risky drinking OR excessive drinking OR problem drinking OR binge drinking OR hazardous drinking OR unsafe drinking OR unhealthy drinking OR drunk OR eating behaviour OR unhealthy eating OR unhealthy diet OR food choice OR food preferences OR food habits OR food intake OR feeding behaviour OR energy intake OR fast foods OR carbonated beverages OR obesity OR overweight OR overnutrition OR overeat OR over-eat OR excess weight OR body size OR body mass OR body weight OR diet OR nutrition OR underweight OR undereat OR under-eat OR undernutrition OR fruit OR vegetable OR portion OR serving OR junk food OR fast food OR processed food OR calorie-dense OR calories OR convenience food OR dietary fat OR dietary sugar OR dietary salt OR fizzy OR sugary OR snack OR takeaway OR takeout OR carry-out OR frozen OR ready-meal
D—Design: qualitative research	Interview OR grounded theory OR ethnography OR interpretative phenomenological analysis OR phenomenology OR focus group OR content analysis OR thematic analysis OR constant comparative OR participant observation OR narratives OR field notes
E—Evaluation: experience	perceive OR perception OR perspective OR view OR experience OR attitude OR belief OR opinion OR feel OR know OR understand
R—Research type: qualitative and mixed methods	Qualitative OR qualitative analysis OR qualitative research OR mixed methods

A sample search strategy for MEDLINE is presented in Table 2. Finally, electronic searches will be supplemented with the following: reference checking of the bibliographies of included studies and key review articles; hand-searching of journals identified as providing the highest yield of references; searches of Google Scholar and topic relevant websites; reference lists already held by reviewers; contact with key researchers in the field.

**Table 2:** Sample MEDLINE search strategy

1. exp. Alcohol Drinking/	
2. exp. alcoholic beverages/	
3. alcoholic intoxication/	
4. (alcohol* adj2 (abuse* or misuse* or use* or consum* or drink* or excess* or problem* or risk*)).mp.	
5. alcohol*.mp.	
6. ((binge or problem* or risk* or excess*) adj2 drink*).mp.	
7. ((hazardous or unsafe or unhealthy) adj2 drink*).mp.	
8. drunk*.mp.	
9. (intoxicat* adj4 (drink* or alcohol*)).mp.	
10. (wine or beer or spirits).mp.	
11. exp. overweight/	
12. exp. overnutrition/or hyperphagia/	
13. exp. “Body Weights and Measures”/	
14. Food preferences/	
15. *Feeding Behaviour/or exp. Food habits/	
16. Energy Intake/	
17. fast foods/or carbonated beverages/	
18. (obes* or over?weight or over?nutrition).ti,ab.	
19. (excess adj2 weight).ti,ab.	
20. ((eat* or food* or feed*) adj2 (behavio?r* or excessive* or choice? or pattern? or habit? or intake? or preference?)).ti,ab.	
21. (body adj2 (mass or size or weight)).ti,ab.	
22. (diet* or nutrition).ti,ab.	
23. over?eat*.mp.	
24. (under?weight or under?nutrition or under?eat).ti,ab.	
25. (unhealth* adj2 (diet* or eating or food*)).mp.	
26. ((vegetable* or fruit) adj2 (eat* or intake or consum* or portion* or serving? or frequenc* or number? or preference? or choice*)).mp.	
27. (((junk or fast or unhealthy or choice? or processed) adj2 food*) or fastfood).mp.	
28. (calorie-dense adj2 (food? or beverage? or drink?)).mp.	
29. (convenien* adj2 (food* or meal*)).mp.	
30. (excess* adj2 (fat* or salt* or sugar*)).mp.	
31. (energy adj1 intake).mp.	
32. (poor adj2 diet).mp.	
33. snack*.mp.	
34. calorie*.mp.	
35. (((fizzy or sugary or carbonated) adj2 drink*) or soda or coca-cola or coke or cola or pop).mp.	
36. (take?away or take?out or carry?out).mp.	
37. (((frozen or ready or TV or television) adj2 meal?) or ((TV or television) adj2 dinner?)).mp.	
38. ((portion or serving) adj2 size?).mp.	
39. or/1-38	
40. Young Adult/	
41. (young adj2 (adult? or person?)).mp.	
42. ((college* or university) adj2 student?).mp.	
43. child*.mp.	
44.under?age*.mp.	
45. late-teen*.mp.	
46. early-adult*.mp.	
47. (adolescen* or youth* or undergraduate* or freshmen or fresher? or teen* or kid* student?).mp.	
48. or/40-47	
49. exp. Interview/or interview.mp.	
50. ‘grounded theory’.mp.	
51. ethnography.mp.	
52. exp. Qualitative Research/or ‘interpretative phenomenological analysis’.mp.	
53. phenomenology.mp.	
54. exp. Focus Groups/or ‘focus group’.mp.	
55. ‘content analysis’.mp.	
56. ‘thematic analysis’.mp.	
57. ‘constant comparative’.mp.	
58. ‘participant observation’.mp.	
59. narrative*.mp.	
60. ‘field notes’.mp.	
61. or/49-60	
62. perceive.mp.	
63. perception*.mp.	
64. perspective*.mp.	
65. view.mp.	
66. experience.mp.	
67. exp. Attitude/or attitude.mp.	
68. belief*.mp.	
69. opinion.mp.	
70. feel*.mp.	
71. know*.mp.	
72. understand*.mp.	
73. or/62-72	
74. exp. Qualitative Research/or qualitative.mp.	
75. ‘qualitative analysis’.mp.	
76. ‘mixed method*’.mp.	
77. or/74-76	
78. 39 and 48 and 61 and 73 and 77	

^*^represents the PubMed symbol for truncation

### Inclusion and exclusion criteria

*Inclusion criteria:*

- Studies reporting primary data of any qualitative design, for example, ethnographic studies, studies that used a phenomenological or grounded theory approach, or participatory action research. Mixed method studies will be considered eligible if findings from qualitative study components are reported in full and can be distinguished from other findings.
- Studies published in English only and from 2006 onwards. This date limit is intended to minimise outdated findings and reflects limited time/resources for the review. It is acknowledged that this decision carries with it a risk of bias.
- Studies that explore the views of young people on factors which shape their eating behaviours or alcohol consumption.
- Studies of individual or groups of young people aged 10–17 (inclusive). This will be determined using age range or mean age at interview. For longitudinal studies, this will be age at recruitment and/or first interview. If results are analysed separately for groups of different ages, and studies include younger children or participants aged 18 or more, only data relating to those aged 10–17 will be extracted. If this data cannot be distinguished from other findings, the study will be excluded. We are particularly interested in exploring the experiences of different socio-demographic groups for example, by age, gender, ethnicity, economic status, or sexual orientation.

*Exclusion criteria:*

- Unpublished data, abstracts, conference proceedings, and studies including no primary evaluation data (e.g. protocols, editorials, reviews).
- Studies that used self-report or researcher-administered surveys, including those which attempt to analyse data from open-ended questions, as the sole method of data collection, as it is felt that survey data cannot explore the topic in sufficient depth.
- Qualitative research that has not ascertained the views young people themselves but has analysed texts, e.g. discourse analysis.
- Studies where the study population: (a) require specialist treatment for alcohol dependency or weight loss and gain (i.e. specialist weight management services (tier 3) and bariatric surgery or drugs (tier 4)); and (b) pregnant or breastfeeding adolescent women whose current eating pattern may be time-limited and not reflective of usual diet behaviours. Those in receipt of tier 1 or tier 2 weight management services or lifestyle interventions will be included in the review.

### Data selection and extraction

The title and abstract of all records retrieved (minus duplicates) will be downloaded to Endnote X7 and independently screened by two researchers, with full text copies of potentially relevant papers retrieved for in-depth review against the inclusion criteria. Any uncertainties will be resolved by discussion and referral to a third party if necessary. Reasons for exclusion will be noted at the full text stage. A flow chart of the selection process, following PRISMA guidelines, will be produced. The JBI Qualitative Assessment and Review Instrument (QARI) [55] will be adapted to an Excel spreadsheet and will guide the extraction of information. Extracted data will include, but not be limited to, phenomena of interest, aims/objectives and methodological approach and analysis methods; conceptual or theoretical basis underlying the study, interviewee characteristics (e.g. sample size, average age, %male/female, education level), and findings of significance to the review question (i.e. influencing factors). Wherever possible, original quotes will be extracted. Data extraction will be carried out by one researcher and checked by another. Where publications lack details required for quality assessment or full data extraction, authors will be contacted to request further information.

### Quality assessment

#### Initial assessment

Identified studies will be assessed by two independent reviewers for methodological validity using the Critical Appraisal Skills Programme (CASP) Qualitative Research Checklist [56]. This appraisal tool was chosen as it can be applied to different types of qualitative design and its 10-item checklist allows rapid and robust evaluation in relation to domains such as appropriateness of study design, data collection techniques, and analysis methods used. Any disagreements that arise between the reviewers will be resolved through discussion or with a third reviewer.

#### Comprehensive assessment

At the second level of appraisal, we will use the GRADE-CERQual guidance [57] to identify which findings are strongly supported or less well supported. No established methods or guidance currently exist on how to assess whether, and to what extent, dissemination bias might be present in the findings of qualitative evidence syntheses [58]. Therefore, we recognise this as a limitation of our assessment of the field. Two reviewers will independently review each study using guidance derived from GRADE-CERQual to reach consensus. In order to facilitate comparisons across the reviewed studies, a table presenting these findings will be presented.

### Data synthesis

Participant quotations and text under the headings “findings” or “results” extracted from identified papers will be entered verbatim into Nvivo 8 software (QSR International, Melbourne, Australia), where data will be stored and coded. Qualitative synthesis will involve three core phases: line-by-line coding of findings, development of descriptive themes, and development of analytical themes [59, 60]. Following the principles of qualitative synthesis, and informed by meta-ethnography in particular, our approach will entail the systematic identification of shared concepts and themes mapped across studies included in the synthesis [61]. We will record key concepts and contextual details to understand the interpretations in every paper, and concepts will be explored for convergent or divergent cases across the studies, by a process referred to as reciprocal translation, similar to the constant comparative techniques used in primary qualitative research. Thus, our synthesis will interpret individual studies by identification of second-order constructs (interpretations offered by the original researchers) and third-order constructs (development of new interpretations beyond those offered in individual studies) by way of the development of a ‘model structure’ of shared influences upon both unhealthy eating behaviours and alcohol use amongst young people aged 10–17 [62]. This model structure will inform subsequent exploratory work and intervention co-design with young people. Meetings will be held to discuss disagreements at any stage of assessment. If unresolved, the opinion of another member of the project team will be sought.

## Discussion

Formative qualitative research, and the synthesis of qualitative work, has a key role to play in intervention development, particularly in relation to exploration of content, design and modality as well as in determining acceptability and feasibility within target population groups [63, 64]. This review is situated within a broader mixed method programme of work. Review findings will first be used to inform in-depth qualitative work which seeks to investigate young people’s viewpoints regarding the relationship between their eating behaviours and linked alcohol use, in order to identify influential factors which cut across both consumption behaviours. Data will subsequently inform the design of tailored, theory-driven multiple risk-behaviour interventions to reduce health and social inequalities due to excess weight gain and alcohol consumption, and thus cancer risk, amongst young people.

## Additional file

Additional file 1: PRISMA-P Checklist. (DOCX 15 kb)

## Abbreviations

ASSIA: Applied Social Sciences Index and Abstracts
BMI: Body mass index
CASP: Critical Appraisal Skills Programme
CINAHL: Cumulative Index to Nursing and Allied Health Literature
ENTREQ: Enhancing transparency in reporting the synthesis of qualitative research
EPPI: Evidence for Policy and Practice Information
ERIC: Education Resources Information Center
JBI: Joanna Briggs Institute
LILACS: Latin American and Caribbean Health Sciences Literature
NAFLD: Non-alcohol fatty liver disease
NCMP: National Child Measurement Programme
NHS: National Health Service
NICE: National Institute for Health and Care Excellence
PRISMA: Preferred Reporting Items of Systematic Reviews and Meta-Analyses
PRISMA-P: Preferred Reporting Items of Systematic Reviews and Meta-Analyses Protocol
QARI: Qualitative Assessment and Review Instrument
